# Computed Tomographic Distinction of Intimal and Medial Calcification in the Intracranial Internal Carotid Artery

**DOI:** 10.1371/journal.pone.0168360

**Published:** 2017-01-06

**Authors:** Remko Kockelkoren, Annelotte Vos, Wim Van Hecke, Aryan Vink, Ronald L. A. W. Bleys, Daphne Verdoorn, Willem P. Th. M. Mali, Jeroen Hendrikse, Huiberdina L. Koek, Pim A. de Jong, Jill B. De Vis

**Affiliations:** 1 Department of Radiology, University Medical Center, Utrecht, Utrecht, The Netherlands; 2 Department of Pathology, University Medical Center, Utrecht, Utrecht, The Netherlands; 3 Department of Anatomy, University Medical Center, Utrecht, Utrecht, The Netherlands; 4 Department of Geriatrics, University Medical Center, Utrecht, Utrecht, The Netherlands; Nagoya University, JAPAN

## Abstract

**Background:**

Intracranial internal carotid artery (iICA) calcification is associated with stroke and is often seen as a proxy of atherosclerosis of the intima. However, it was recently shown that these calcifications are predominantly located in the tunica media and internal elastic lamina (medial calcification). Intimal and medial calcifications are thought to have a different pathogenesis and clinical consequences and can only be distinguished through ex vivo histological analysis. Therefore, our aim was to develop CT scoring method to distinguish intimal and medial iICA calcification in vivo.

**Methods:**

First, in both iICAs of 16 cerebral autopsy patients the intimal and/or medial calcification area was histologically assessed (142 slides). Brain CT images of these patients were matched to the corresponding histological slides to develop a CT score that determines intimal or medial calcification dominance. Second, performance of the CT score was assessed in these 16 patients. Third, reproducibility was tested in a separate cohort.

**Results:**

First, CT features of the score were circularity (absent, dot(s), <90°, 90–270° or 270–360°), thickness (absent, ≥1.5mm, or <1.5mm), and morphology (indistinguishable, irregular/patchy or continuous). A high sum of features represented medial and a lower sum intimal calcifications. Second, in the 16 patients the concordance between the CT score and the dominant calcification type was reasonable. Third, the score showed good reproducibility (kappa: 0.72 proportion of agreement: 0.82) between the categories intimal, medial or absent/indistinguishable.

**Conclusions:**

The developed CT score shows good reproducibility and can differentiate reasonably well between intimal and medial calcification dominance in the iICA, allowing for further (epidemiological) studies on iICA calcification.

## Introduction

Calcification of the intracranial internal carotid artery (iICA) on Computed Tomography (CT) is an independent predictor of stroke in the general white population and was associated with 75% of all stroke in the Rotterdam study.[1] Calcifications of the iICA, commonly referred to as the carotid siphon due to the tortuous shape, have also been associated with lacunar infarctions[2,3] and white matter hyperintensities on MRI.[4]

While calcifications in the iICA are often seen as a proxy of atherosclerotic burden and thereby are thought to be situated in the arterial intimal layer, a recent histology study showed that these calcifications are predominantly non-atherosclerotic and are located in the tunica media and around the internal elastic lamina.[5] Because calcifications of the medial layer of the vascular wall and calcifications around the internal elastic lamina are thought to be related and are therefore from here on grouped as medial calcifications.[6]

Calcification of the arterial intimal and medial layer are presumed to have a different pathogenesis and clinical consequences.[7] The intimal layer consists of endothelial cells that ‘proliferate’ in the process of atherosclerosis growing into the arterial lumen forming plaques that narrow the lumen and can rupture, causing thromboembolic events.[8] Whereas the medial layer consists of smooth muscle cells and elastic fibers which have a function in regulating blood flow and arterial pressure. Calcification of the media is thought to cause stiffening, reduce compliance and limit distensibility. This can cause an increase in pulse wave velocity and pulse pressure and subsequently chronic damage to the brain tissue.[9,10] Distinction of these calcification types in the iICA would be desirable to further investigate their respective roles in (neuro)vascular disease.

Ex vivo histological analysis is the gold standard to distinguish intimal and medial calcification.[7] Studies that differentiate both calcification types in vivo using either physiological tests (high ankle-brachial index), X-ray or ultrasound have been carried out but the literature in this field is sparse. Most studies are focused on the lower extremities and breast arteries due to technical limitations of visualizing arteries deeper in the body, especially in the head.[11,12] Hence there is no valid in vivo method to separate iICA calcifications while the distinction between both could prove valuable.

Therefore, our aim was to develop a scoring method which distinguishes intimal from medial iICA calcifications in vivo to enable further epidemiological studies on this subject. For this purpose, CT imaging was preferred based on its ability to visualize arterial calcifications and its wide use in clinical practice. A comparison between histology data and CT imaging characteristics was performed per patient to develop a visual CT score which could determine intimal or medial calcification dominance in the iICA. Subsequently, performance and reproducibility of the score was determined.

## Methods

### Study Design

Our study consisted of three steps. In the first and second step we developed and validated the CT score in a single cohort. In the third step we assessed the reproducibility of the score in a separate cohort which will be described later. For the first two steps we included 16 consecutive deceased patients in whom cerebral autopsy was performed between April 2014 and February 2015. The inclusion criteria was a brain CT examination 6 months prior to autopsy. All patients had a routine autopsy for which consent was given by their next of kin. Permission for the evaluation of rest material from the autopsies was given by the local Biobank Review Committe, under protocol number 15–252. Material was handled in a coded way that met the criteria of the code of proper use of human tissue, used in The Netherlands. Informed consent for data used in assessing reproducibility was waived by the Medical Ethical Testing Committee (METC) due to the retrospective nature of the study (approval number: 16/092).

### Histological Analysis

After removal of the brain, the iICA was dissected as close to the petrous bone as possible and removed. Subsequently the arteries were fixed in 4% formaldehyde, and (partially) decalcified using diaminoethylene tetraacetic solution (EDTA). Decalcification was necessary to maintain the morphology of the vascular wall during tissue processing, and does not influence the analyses since the matrix previously altered by the calcification process still remains.[13]. The arteries were divided in a proximal (C4 and C5 according to the classification system proposed by Bouthillier) and a distal (C6) segment (S1 Fig),[14] the intersection was perpendicular to the lumen Per segment, 2–3 histological slides, stained with hematoxylin and eosin and elastin van Giesson, were used to digitally analyse the total surface of calcifications in the intima, in the media and around the internal elastic lamina, as previously described.[5] Calcifications were characterized by quite sharp demarcated, acellular spots and areas, which were dark pink to purple colored on hematoxylin and eosin stained slides. For this study digital images of 142 slides were available.

Dominant calcification type was determined per patient by adding the calcification areas in all slides to a summed intimal and medial calcification burden. If the summed area of medial calcification was larger than the summed area of intimal calcification the patient was categorized by histology as medial dominant and vice versa.

### CT Image Acquisition and Analysis

The 16 patients were scanned on a Philips Brilliance 64-slice or 256-slice CT scanner (Philips Healthcare, Best, The Netherlands) from the skull base to the vertex. Tube voltage was either 120kVp or 140kVp and tube current ranged between 200–250 mAs. For adequate detection of the subtle/thin calcifications only non-contrast enhanced CT’s were used with slice thickness between 0,625 and 1mm. The image quality was assessed and all images were deemed to be of good/adequate quality without evident artefacts (beam hardening, photon starvation, noise) that could potentially influence image evaluation. Images were assessed in bone setting (Center: 300 Hounsfield Units–Width: 1600 Hounsfield Units) in all planes (axial, sagittal and coronal).

**Fig 1:**
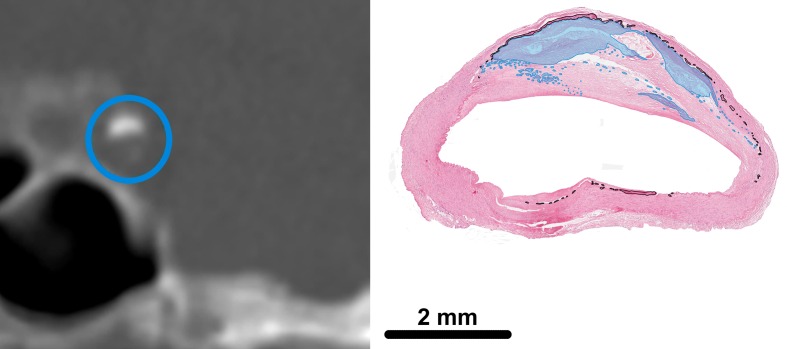
Intimal calcification in the intracranial internal carotid artery (iICA) on a coronal brain CT image (left) and on a histological slide (right). On CT a blue circle is placed around the iICA. In histology the intimal calcification area is light blue and the calcification area of the internal elastic lamina indicated by the black line.

**Fig 2:**
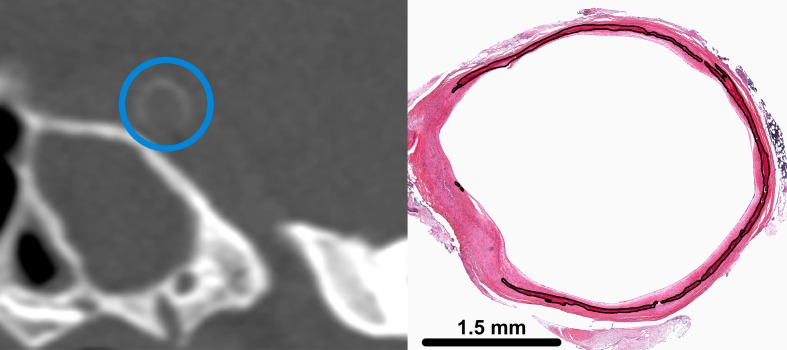
Internal elastic lamina calcification in the intracranial internal carotid artery (iICA) on a coronal brain CT image (left) and on a histological slide (right). On CT a blue circle is placed around the iICA. Calcification area of the internal elastic lamina is indicated by the black line. Reprinted from A. Vos et al. Stroke. 2016;47:221–223 (Fig 1A) under a CC BY license, with permission of the American Heart Association, original copyright 2016 American Heart Association.

Calcifications in the iICA on CT were analysed in concordance with the 142 histological slides. The location of the histological slide in the iICA was registered and with this information the corresponding CT slide could be approximated by using multiplanar reconstruction in any direction. Both the histological slide and corresponding CT image were then displayed side by side to allow for adequate comparison (Figs 1 and 2).

### Calcification Score: Development

In step 1 histological and CT slices were compared to develop a visual calcification score. For this, emphasis was put on distinctive features of intimal and medial calcification, as earlier described in literature.[7] I.e. medial calcifications have been described as being often thin and progress in a circular pattern while intimal calcifications (that co-localize with atherosclerotic plaques) are more clustered and often grow intraluminal.[15,16]

To develop the score, points were assigned to the different calcification characteristics found in the analysis of the matched CT- and histological slides. The points awarded per characteristic were weighted according to their relation to either medial or intimal calcification. Subsequently, for each combination of characteristics it was decided whether it was associated with dominant intimal or dominant medial calcifications and based on all combinations an optimal point threshold for dividing calcification dominance was determined. If a calcification was visible on CT, but the amount was too limited to assign any morphological characteristics to, it was classified as indistinguishable.

### Calcification Score: Performance

In step 2 the performance of the developed calcification CT score was evaluated in the 16 autopsy patients by two raters (P.A.D.J. and J.B.D.V.) with respectively fourteen and three years of experience in reading CT scans and who were not involved in the development of the CT score. Both raters were blinded for the histological reference standard. The final score of either dominant intimal, medial or indistinguishable/absent as rated with the CT score was compared to the results of the dominant calcification type as was determined by histology.

### Calcification Score: Reproducibility

To assess the applicability of the score the interrater reliability and agreement were determined. For that purpose a larger sample size was preferred. Therefore in step 3 we randomly selected CT scans from 48 patients who were part of a larger study of patients with a suspected acute stroke.[17] To determine applicability of the score in the general population and compare the reproducibility to (suspected) stroke patients we matched patients from an ongoing study on trauma patients by age and gender to the 48 stroke patients. Trauma patients were chosen as reference over other patient groups as they represent a random sample of the general population due to the random nature of traumas. Two raters (J.B.D.V. and R.K) with respectively three and two years of experience in reading CT scans independently scored the 96 CT scans.

### Statistical Analysis

For the CT-histology performance, three by three tables were drawn and diagnostic characteristics were determined. For the interrater reliability and agreement study kappa values, with 95% confidence intervals, and proportions of agreement were calculated, respectively. Kappa’s of circularity and thickness were linearly weighted as these are ordinal measures. A Kappa value of >0.60 was regarded as good reliability. Data analysis was performed with R (R Foundation for Statistical Computing, Vienna, Austria. https://www.R-project.org/).

## Results

The median age of the 16 patients (ten males, six females) was 64 years (range 44–85). Two of the patients were diagnosed with diabetes mellitus type 2 and one patient was known to have kidney disease. In 11 patients intracranial pathology was found; tumor (2), hemorrhage (5), and ischemia (4). Dominant calcification types of all histology slides are presented in Table 1.

**Table 1:** Distribution of calcification dominance per histological slide.

	Location	Medial	Intimal
**Left iICA** n, (%)	**Proximal**	23 (62)	9 (24)
	**Distal**	25 (81)	5 (16)
**Right iICA** n, (%)	**Proximal**	34 (85)	4 (10)
	**Distal**	29 (85)	5 (15)
**Total**		111(78)	23 (16)

iICA: intracranial internal carotid artery, n = number, Proximal = C4-C5 and Distal = C6 (Classification of Bouthillier[14])

### Development

**Fig 3:**
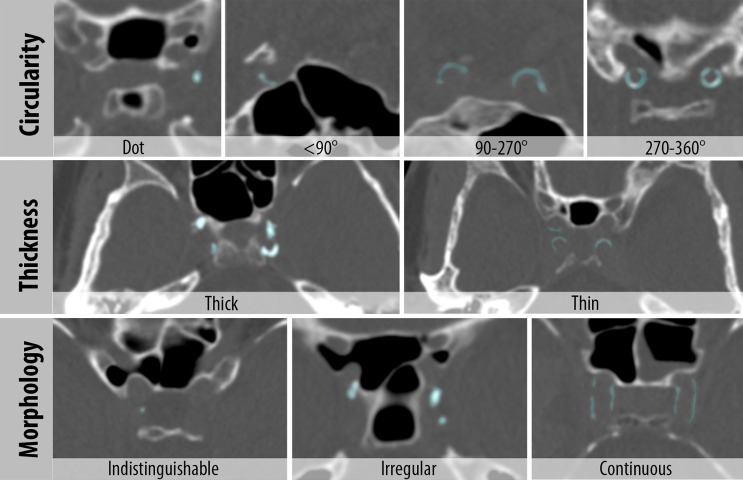
Intracranial internal carotid artery calcification (iICA) score with Circularity (Dot, <90°, 90–270° and 270–360°); Thickness (Thick ≥ 1.5mm and Thin < 1.5mm) and Morphology (Indistinguishable, Irregular, Continuous). Calcifications are highlighted (light blue). In these examples all images are in the axial viewing plane except for the <90° and 90–270° images which are in the coronal plane.

The analysis of the matched histological and CT slices showed that, in accordance with previous literature, intimal calcifications were overall clustered, thick and scattered/patchy throughout the artery (Fig 1) whereas medial calcifications were overall circular, thin and continuous (Fig 2). Based on these findings the characteristics for the score were determined as circularity, thickness and morphology. The final visual score was as follows (Fig 3, Table 2):

**Table 2:** Intracranial internal carotid artery calcification score for unenhanced CT.

Characteristic		Points
**Circularity**	Absent	0
	Dot(s)	1
	<90 degrees	2
	90–270 degrees	3
	270–360 degrees	4
**Thickness**	Absent	0
	Thick ≥ 1.5mm	1
	Thin < 1.5mm	3
**Morphology**	Indistinguishable	0
	Irregular/Patchy	1
	Continuous	4

<7: Dominant Intimal, ≥ 7: Dominant Non-Intimal

Calcification circularity was scored from absent (0 points) to 270–360 degrees (4 points) based on the most circular part in a perpendicular viewing plane. The maximal circularity within the iICA is used for the score.

Calcification thickness was scored as either thick (1 point) or thin (3 points). If the thickest calcification exceeded 1.5mm, measured perpendicular to the arterial wall, the calcifications were scored as thick. The maximum thickness of both iICA’s was used for the score.

Calcification morphology could either be indistinguishable (0 points), irregular/patchy (1 point) or continuous (4 points). Indistinguishable was used to describe dot like calcifications that were too small to assign any morphological characteristics to, and were therefore not categorizable. Irregular and/or patchy referred to spread out or irregular calcifications. Continuous calcifications were regular calcifications spread over a longer arterial segment. The dominant morphology pattern over the trajectory of the iICAs was scored.

Based on all possible combinations of points a threshold of seven was determined as the optimal value to separate intimal and medial calcifications (S1 Table). If after adding up the points the total was lower than seven, the calcifications were thought to be dominantly intimal, whereas if the total was over or equal to seven the calcifications were thought to be dominantly medial. An exception holds for calcifications that were deemed indistinguishable. Furthermore, the CT score determined the dominant calcification type on a patient basis instead of for each iICA separately as on histological slides the calcification pattern was symmetrical in 88% of patients. The score was applied accordingly for determining performance and reproducibility.

### Performance

Performance of the CT score can be found in Table 3. Rater 1 classified eight patients correctly, four indistinguishable/absent and four incorrectly. Rater 2 classified nine patients correctly, three indistinguishable/absent and four incorrectly. When accounting for the indistinguishable calcifications, the performance of the score was reasonable. Three misclassified iICA calcifications were found in both raters. The majority of incorrectly classified patients had little iICA calcification which made them harder to classify. Most notable was one patient in which dominant intimal calcification was scored as dominant medial calcification because of the more circular nature of the intimal calcifications in this particular patient. This was the only patient in our sample showing intimal calcification with high circularity. In the other two, dominant medial calcification was scored as dominant intimal calcification. On the CT images multiple dot-like calcifications were present whereas the medial calcification that was visible on the histological slides was too subtle to detect on CT. As another example of the detectability of these calcifications: in the three patients where both raters scored absent, medial calcifications were present that could not be detected due to a limited detection threshold of CT.

**Table 3:** Performance of the calcification score in the intracranial internal carotid artery.

	Rater 1	Model				Rater 2	Model			
		Medial	Intimal	Absent*	Total		Medial	Intimal	Absent*	Total
**Histology**	**Medial**	5	2	4	11	**Medial**	6	3	3	12
	**Intimal**	2	3	0	5	**Intimal**	1	3	0	4
	**Absent**	0	0	0	0	**Absent**	0	0	0	0
	**Total**	7	5	4	16	**Total**	7	6	3	16

*or indistinguishable

### Reproducibility

The median age of the 96 scored patients (58% male) was 69 (range 41–90) years. Results of the CT score by both readers were as follows (S2 and S3 Tables). Reproducibility results can be found in Table 4. Reliability between the raters was 0.80 (CI: 0.72–0.88) for circularity, 0.75 (CI: 0.65–0.86) for thickness and 0.70 (CI: 0.57–0.82) for morphology. The proportion of agreement between the raters was 0.74 (CI: 0.64–0.82) for circularity, 0.81 (0.72–0.88) for thickness and 0.80 (0.71–0.87) for morphology. Reliability and agreement for the overall score was 0.72 (CI: 0.60–0.84) and 0.82 (CI: 0.73–0.89). Both reproducibility and agreement were comparable between the stroke and reference cohort (Table 4).

**Table 4:** Reproducibility of the calcification score.

		Full cohort (n = 96)	Stroke cohort (n = 48)	Reference cohort (n = 48)
Interrater reliability*	**Circularity**	0.80 (0.72–0.88)	0.77 (0.65–0.89)	0.82 (0.72–0.92)
	**Thickness**	0.75 (0.65–0.86)	0.76 (0.61–0.90)	0.75 (0.60–0.91)
	**Morphology**	0.70 (0.57–0.82)	0.65 (0.46–0.83)	0.74 (0.58–0.91)
	**Calcification score**	0.72 (0.60–0.84)	0.71 (0.53–0.88)	0.73 (0.57–0.90)
**Interrater agreement**	**Circularity**	0.74 (0.64–0.82)	0.73 (0.58–0.84)	0.77 (0.62–0.87)
	**Thickness**	0.81 (0.72–0.88)	0.81 (0.67–0.91)	0.81 (0.67–0.91)
	**Morphology**	0.80 (0.71–0.87)	0.77 (0.62–0.88)	0.83 (0.69–0.92)
	**Calcification score**	0.82 (0.73–0.89)	0.81 (0.67–0.90)	0.83 (0.69–0.92)

*Kappa

## Discussion

In this study we showed that a reasonable distinction can be made in vivo between intimal and medial calcification dominance in the iICA. We developed a CT score that can be used on non-contrast enhanced CT images and has good agreement between raters.

Calcification of the iICA on CT images in relation to intracranial pathology has been studied extensively using both visual and quantitative scores. Visual scores provide an overall good, quick and reproducible measure of iICA calcification burden[18] whereas quantitative or volumetric calcification measurement are time intensive but do provide a more accurate (volumetric) depiction of the calcium load/burden.[1] However, the previous qualitative scores were not designed to separate intimal and medial calcification. The score developed in this study combines the visual characteristics circularity, thickness and morphology, as previously used in iICA calcification scores.[3,4,19–21] Our comparison with histology showed that these described characteristics, after some adaptation, were a good basis to separate intimal and medial calcifications.

The distinction of intimal from medial calcification could be of importance for several reasons. One, calcification of the iICA is related to stroke and other intracranial vascular diseases, but it is unclear whether this is attributable to intimal calcifications, medial calcifications or a combination of both. Also the etiology (and ultimately treatment) of dominant intimal or medial disease may be different. Two, in case of medial calcification the compliance of the arterial wall may be diminished leading to an increased pulsatility and thereby pulse wave velocity.[22,23] Medial calcification of the iICA may therefore explain the observed associations between iICA calcification with white matter hyperintensities[4] and acute small vessel infarcts.[3] Three, medial calcification was found to comprise 71% of the dominant calcification burden and thereby it’s influence on physiopathology may be underestimated.[5] Four, clinically medial calcification is mostly thought to be a passive and chronic process, however, in a recent review it was shown that it is actually an active process resembling bone formation.[7] The limited knowledge on the clinical consequences and therapeutic options for medial calcification mainly comes from genetic syndromes and epidemiological studies outside the brain. Our CT score provides the opportunity to further investigate the role of medial calcification in cerebrovascular diseases.

The data presented in this paper demonstrated that the majority of iICAs were either scored correctly according to their dominant calcification pattern as visualized on histology, or were scored as indistinguishable or absent. When calcification was absent on CT images, it was at times still detected on histology in very minute amounts but remained under the detection threshold of CT. The indistinguishable category represents patients with too little visible calcification on CT images to be properly classified. Most likely, due to their limited amount of calcification, those patients are also the ones who least benefit from early intervention and thereby a lack of classification could be considered to be less of importance within this group of patients. The remaining 25% of our iICAs were classified incorrectly. Interestingly, the majority of these patients also had little amount of calcification on the CT images and thereby could have been on the edge of what is reasonable to classify. Adequate stratification of these calcifications will probably remain problematic with the current resolution of CT scanners.

A major strength of this study is that we used histology as a reference standard. Often CT scores are developed without a solid reference standard. Another strength of this study is the use of good quality CT data with thin slices (0.625–1 mm) and multiplanar reconstructions to evaluate the calcifications. Intracranial calcification is often scored on a slice thickness of 5mm and on axial plane only. Considering that the cavernous iICA is on average 4-7mm in diameter and often runs parallel on axial images, the amount of slices suitable for scoring is limited. Also, evaluating the circumferential degree of calcification on thick axial slices can be quite problematic. For these reasons, we would also recommend future studies who are implementing the presented CT score, to evaluate thin slices and make use of multiplanar reconstructions.

There are some limitations to this study. First, even though we attempted to make an optimal comparison between histology and CT we cannot be entirely certain that the CT images exactly represented the histological slides. However, we do think that our 142 comparisons gave us overall a good impression of the morphological differences in medial and intimal calcification on CT. Second, the performance was evaluated on a limited sample size of 16 patients. The histological analysis of the calcifications is time consuming (all individual calcification deposits are manually scored) which limited us in the number of samples we were able to analyse. Third, strokes of cardioembolic origin will not be captured by the calcification score. This could influence results in analysis of iICA calcification and stroke etiology and should therefore always be considered. Lastly, although we did find reasonable performance in a labour intense and therefore small study and good reproducibility between two raters in a larger sample, further investigations are needed before the CT score can be applied as a diagnostic tool in patients. Nevertheless, the presented score could already be used in epidemiological and genetic studies investigating the clinical implications of medial calcification.

### Conclusion

In conclusion, we developed a reproducible CT based scoring tool that can reasonably distinguish intimal and medial calcification dominance in the intracranial internal carotid artery. This CT score could be used for future in vivo epidemiological studies to better understand the role of intimal and medial calcification at this location of the arterial system. The ability to separate these different forms of calcifications and the improved phenotyping of iICA disease in vivo could help to better understand cerebrovascular diseases.

## Supporting Information

S1 Fig Schematic of the intracranial carotid artery and subsequent cerebral arteries. Histology of calcifications was analysed per patient both proximal (C4-C5) and Distal (C6). a: Anterior cerebral artery; b: Middle cerebral artery; c: Posterior cerebral artery; d: Posterior communicating artery; e: Ophthalmic artery; Pink: C7; Green: C6; Blue: C5; Yellow: C4 (cavernous sinus); *anterior clinoid process; ** carotid canal and foramen lacerum.
(DOCX)

S1 Table Combinations of calcification characteristic points and calcification association. (DOCX)

S2 Table Calcification score results in 48 patients with suspected stroke. (DOCX)

S3 Table Calcification score results in 48 trauma patients. (DOCX)
