# Supplementary material for: Computed Tomographic Distinction of Intimal and Medial Calcification in the Intracranial Internal Carotid Artery
Source: PLoS One. 2017 Jan 6;12(1):e0168360. doi: 10.1371/journal.pone.0168360 (PMC5218397; doi:10.1371/journal.pone.0168360)
Supplement: S1 Fig — Histology of calcifications was analysed per patient both proximal (C4-C5) and Distal (C6). a: Anterior cerebral artery; b: Middle cerebral artery; c: Posterior cerebral artery; d: Posterior communicating artery; e: Ophthalmic artery; Pink: C7; Green: C6; Blue: C5; Yellow: C4 (cavernous sinus); *anterior clinoid process; ** carotid canal and foramen lacerum. (DOCX) [file pone.0168360.s001.docx]

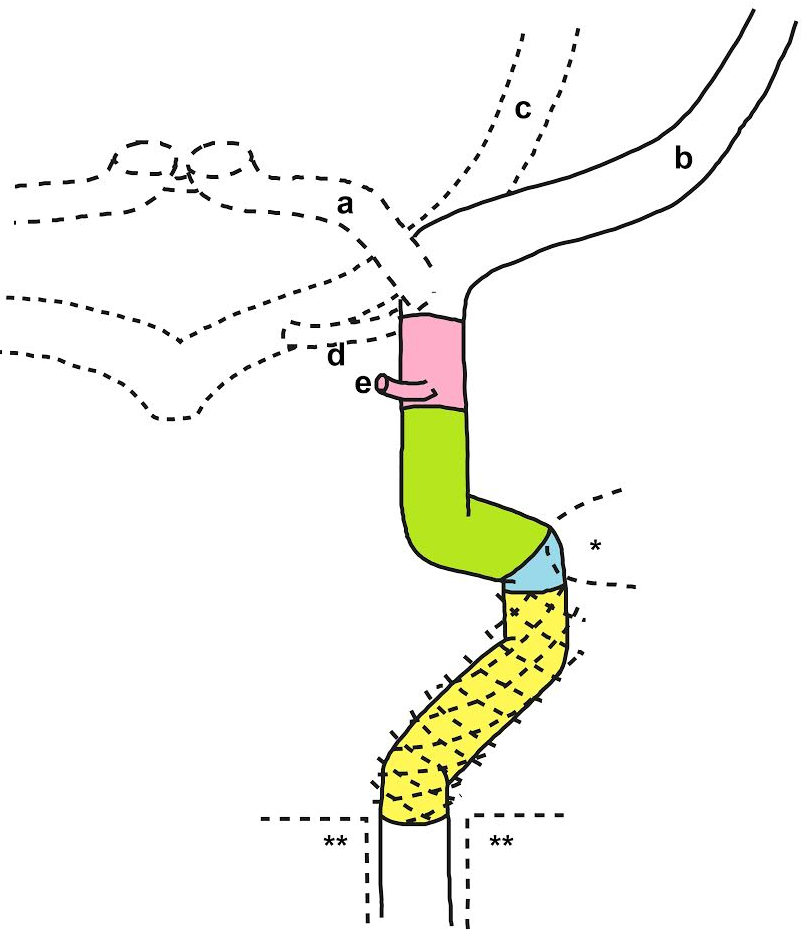


**S1 Fig.** **Schematic of the intracranial carotid artery and subsequent cerebral arteries.** Histology of calcifications was analysed per patient both proximal (C4-C5) and Distal (C6).

a: Anterior cerebral artery; b: Middle cerebral artery; c: Posterior cerebral artery; d: Posterior communicating artery; e: Ophthalmic artery; Pink: C7; Green: C6; Blue: C5; Yellow: C4 (cavernous sinus); *anterior clinoid process; ** carotid canal and foramen lacerum.
