# Supplementary material for: Computed Tomographic Distinction of Intimal and Medial Calcification in the Intracranial Internal Carotid Artery
Source: PLoS One. 2017 Jan 6;12(1):e0168360. doi: 10.1371/journal.pone.0168360 (PMC5218397; doi:10.1371/journal.pone.0168360)
Supplement: S1 Table — (DOCX) [file pone.0168360.s002.docx]

| **S1 Table. Combinations of calcification characteristic points and calcification association** | | | | |
| --- | --- | --- | --- | --- |
| **Points** | **Circularity** | **Thickness** | **Morphology** | **Dominance** |
| **2** | Dot | Thick | Indistinguishable | Intimal* |
| **3** | Dot | Thick | Irregular |  |
| **4** | Dot | Thin | Indistinguishable |  |
|  | <90° | Thick | Irregular |  |
| **5** | Dot | Thin | Irregular |  |
|  | 90-270° | Thick | Irregular |  |
| **6** | <90° | Thin | Irregular |  |
|  | 270-360° | Thick | Irregular |  |
| **7** | <90° | Thick | Continuous† | Medial |
|  | 90-270° | Thin | Irregular |  |
| **8** | 270-360° | Thin | Irregular |  |
|  | 90-270° | Thick | Continuous |  |
| **9** | <90° | Thin | Continuous |  |
|  | 270-360° | Thick | Continuous |  |
| **10** | 90-270° | Thin | Continuous |  |
| **11** | 270-360° | Thin | Continuous |  |
| = Associated with intimal calcification  = Associated with medial calcification  = Unassociated  *Except for indistinguishable calcifications, † Did not occur in our CT images. | | | | |
