# Supplementary material for: Computed Tomographic Distinction of Intimal and Medial Calcification in the Intracranial Internal Carotid Artery
Source: PLoS One. 2017 Jan 6;12(1):e0168360. doi: 10.1371/journal.pone.0168360 (PMC5218397; doi:10.1371/journal.pone.0168360)
Supplement: S3 Table — (DOCX) [file pone.0168360.s004.docx]

| **S3 Table. Calcification score results in 48 trauma patients** | | | | |
| --- | --- | --- | --- | --- |
|  | | **Rater 1** | | |
|  |  | **Medial** | **Intimal** | **Absent*** |
| **Rater 2** | **Medial** | 21 | 0 | 0 |
|  | **Intimal** | 6 | 8 | 0 |
|  | **Absent*** | 0 | 2 | 11 |
| *or indistinguishable | | | | |
